# Glucose Supplementation Improves Performance and Alters Glucose Transporters’ Expression in *Pectoralis major* of Heat-Stressed Chickens

**DOI:** 10.3390/ani13182911

**Published:** 2023-09-14

**Authors:** Oluwatomide Williams Ariyo, Josephine Kwakye, Selorm Sovi, Bikash Aryal, Ahmed F. A. Ghareeb, Evan Hartono, Marie C. Milfort, Alberta L. Fuller, Romdhane Rekaya, Samuel E. Aggrey

**Affiliations:** 1 NutriGenomics Laboratory, Department of Poultry Science, University of Georgia, Athens, GA 30602, USA; oluwatomide.ariyo@uga.edu (O.W.A.); josephine.kwakye@uga.edu (J.K.); selorm.sovi@uga.edu (S.S.); bikash.aryal@uga.edu (B.A.); ahmed.ghareeb@uga.edu (A.F.A.G.); evan.hartono@uga.edu (E.H.); marie.milfort@uga.edu (M.C.M.); alfuller@uga.edu (A.L.F.); 2 Department of Animal and Dairy Science, University of Georgia, Athens, GA 30602, USA; rrekaya@uga.edu

**Keywords:** heat stress, glucose supplementation, performance, carcass yield, glucose transporters

## Abstract

**Simple Summary:**

Heat stress (HS) is a major challenge in poultry production as it alters basic metabolism and slows growth. The reduction in feed intake (FI), induced by heat stress is a major determinant as it reduces glucose intake and level in the tissues of broiler chickens. Reduced tissue glucose level causes a reduction in the energy level of birds, hence increasing gluconeogenesis (i.e., utilizing muscle protein to produce glucose). This results in poor carcass yield and economic losses. We aimed at sustaining tissue glucose of birds subjected to cyclic HS via glucose supplementation. Heat stress decreased the performance of the birds while glucose supplementation salvaged some of the losses that could have been incurred under HS. The expression of SGLT1 was pronounced in the glucose supplemented groups while GLUTs 1, 5 and 10 were highly expressed in birds subjected to HS. Sustaining tissue glucose level of birds may aid in the mitigation of some of the detrimental effects of HS.

**Abstract:**

Glucose level in birds’ tissue decreases due to heat stress (HS)-induced reduction in feed intake (FI); impairing metabolism and growth. The effect of glucose supplementation on the performance of broiler chickens was evaluated under thermoneutral (TN) and HS conditions. Glucose was supplemented at 0 and 6% under TN-(25 °C) and HS-(25 °C–35 °C–25 °C) conditions. The treatments were TN + 0%-glucose (TN0); TN + 6%-glucose (TN6), HS + 0%-glucose (HS0) and HS + 6%-glucose (HS6). There were 6 replicates (19 birds each)/treatment. Heat and glucose supplementation were applied from d28–35. At d35, *Pectoralis (P.) major* was sampled from one bird/replicate to determine glucose transporters’ mRNA expression. Heat application lowered (*p* < 0.05) FI, body weight gain, and increased feed and water conversion ratios. Glucose supplementation increased total energy intake by 4.9 and 3.2% in TN and HS groups, respectively but reduced FI under TN and HS conditions. The *P. major*- and drumstick-yield reduced (*p* < 0.05) in HS0 compared to TN0, TN6 and HS6. Under HS, glucose supplementation improved eviscerated carcass weight by 9% and *P. major* yield by 14%. Glucose supplementation increased SGLT1 expression with/without heat treatment while HS independently increased the expression of GLUT 1, 5 and 10. Glucose supplementation under HS could improve performance of broilers.

## 1. Introduction

Heat stress (HS) occurs when metabolic heat produced within the body of an animal exceeds the amount of heat dissipated [1,2]. This is pronounced in areas with high ambient temperature and humidity, such as tropics, subtropics, and summer period in temperate regions [3,4,5]. Heat stress can be acute (short-lived) or chronic (long-lasting); both negatively impacting the health and performance of poultry [2,5,6,7]. Modern commercial broiler strains are more vulnerable to HS due to their high metabolic rate caused by genetic selection for meat production [8,9,10]. The lack of sweat glands also increases heat dissipation inefficiencies in chickens subjected to HS [11,12,13,14], making HS a major concern in poultry production.

Animals exposed to HS exhibit a variety of physiological responses, such as reduced feed intake, elevated rectal temperature, accelerated heart and respiration rates, and excessive water consumption [14]. The reduced feed intake frequently results in a higher feed conversion ratio and reduced weight gain [15]. Heat stress alters carcass yield and quality [16] due to protein catabolism and oxidative damage in muscles [2]. This impairment may change the levels of different enzymes, antioxidants, and metabolites such as blood glucose. An increase in the level of circulating glucose due to high circulating corticosterone induced by HS has been reported [17,18]. On the other hand, the level of tissue glucose declines with reduced feed intake due to HS [19], suggesting that glucose metabolism is altered under HS.

Glucose is required as fuel for all metabolic processes in the cells of all animals [20,21]. To survive under HS, birds resort to meet energy requirements by generating glucose from pyruvate and other non-glucose sources (e.g., muscle protein) within the body [22,23]. Protein is the primary component of chickens’ growth [24]. Hence, utilization of muscle proteins for energy will impair growth that could ultimately lead to financial losses [25]. It is therefore pertinent to develop strategies that would ameliorate the adverse effect of HS in broiler chickens.

Several nutritional approaches such as the use of feed additives (amino acids, fatty acids, minerals, vitamins, probiotics, electrolytes) and feed restriction have been employed to mitigate HS in broiler chickens [26,27] but none has directly addressed the possibility of sustaining tissues’ glucose level while evaluating the role of glucose transporters in the breast muscle of the birds. This could curtail the use of muscle protein as a source of energy under HS condition. We hereby hypothesize that the supplementation of glucose would improve tissue glucose levels through the changes in the molecular expression of glucose transporters and improve the performance broiler chickens raised under HS condition.

## 2. Materials and Methods

### 2.1. Animals and Diets

This study was carried out using 456 Cobb500 mixed sex broiler chickens; randomly allocated to 4 treatment groups with 6 replicates containing 19 birds each. Prior to assigning birds to the various treatment groups, birds were reared on fresh wood shavings from one-day old until 28 d. All the birds were fed a starter diet from hatch to 14 days, a grower diet from 14–28 days and a finisher diet from day 28–35 days. The starter diet comprised of 22.21% crude protein (CP) and 2980 ME/Kcal/kg, grower diet comprised of 20.15% CP and 3075 ME/Kcal/kg and the finisher diet comprised of 18.26% CP and 3125 ME/Kcal/kg. The birds received water and feed *ad libitum*. The pens were individually equipped with feeding troughs and known quantity of feed were supplied to the birds to monitor feed intake. Each pen had a well calibrated container connected to it to measure water consumption. Heat application and glucose supplementation started at the finisher phase from 28–35 days of age. The birds were raised under thermoneutral (TN) conditions (25 °C) or cyclical HS conditions (35 °C from 8 a.m. to 8 p.m. and 25 °C from 8 p.m. to 8 a.m.). Within each temperature group, half of the birds had no glucose supplementation and the other half had 6% D glucose in their drinking water. The experimental period started on day 28 which corresponds to the beginning of the finisher phase. Broiler chickens are prone to HS at this phase more than the earlier phases. The four treatment groups were: TN with 0% glucose supplementation (TN0), TN with 6% glucose supplementation (TN6), HS with 0% glucose supplementation (HS0) and HS with 6% glucose supplementation. All animals regardless of treatment were fed the same diet. Replicates were randomly assigned within each temperature treatment. In all, there were 114 birds in each treatment group (6 replicates and 19 birds per replicate). Each replicate consisting of 19 birds were raised in a pen (152.4 cm long, 121.92 cm wide, and 60.96 cm high).

### 2.2. Growth Performance

Body weight (BW) at day 28 and 35, feed intake (FI) and water intake (WI) per pen were measured at 28–35 days. Feed intake and water intake were calculated as the difference in the amount given and the left over. From these measurements, we calculated the BW gain (BWG) as the difference between BW at day 28 and 35, feed conversion ratio (FCR) as the proportion of feed intake to weight gain between day 28 and 35 and water conversion ratio (WCR) for each pen as the proportion of feed intake to weight gain between day 28 and 35. The metabolizable energy (ME) intake was calculated as the total ME obtained from feed and water consumed by the birds.

### 2.3. Carcass Yield

Three birds per pen (n = 18/treatment) were randomly selected and processed at the University of Georgia Processing Plant (Athens, GA, USA) on day 7 post-HS. The birds were humanely stunned via electrocution at 25 V and 25 mA, slaughtered, and moved through the defeathering machine. The head, shanks and offal were removed. The remaining carcass (empty carcass) was thoroughly rinsed and chilled at 4 °C for 24 h. The carcasses were eviscerated and the *Pectoralis* (*P.*) *major*, *P. minor*, thigh, drumstick, and wing and abdominal fat weights were recorded. The carcass yield (CY) was calculated as a percentage of empty carcass weight of the BW prior to processing. The *P. major*- (PMY), *P. minor* (PMinY)-, thigh (TY)-, drumstick (DY)- wing (WY)- and abdominal fat yield were calculated as percentage of their respective weights and the empty carcass weight.

### 2.4. Tissue Collection

On day 35, one bird per pen was randomly sampled. Birds were humanely euthanized by cervical dislocation and a portion of the *P. major* was taken, snap frozen in liquid nitrogen. The samples were transferred to a −86 °C freezer until further analysis.

### 2.5. Gene Expression of Glucose Transporters

Total RNA was extracted using Trizol method (Invitrogen, Carlsbad, CA, USA). The extracted RNA was cleaned up using RNeasy Mini Kit (Qiagen, Valencia, CA, USA) following the procedure outlined by the manufacturer. The concentration of clean RNA was measured with NanoDrop 2000 Spectrophotometer (Thermo Scientific, Wilmington, DE, USA) and adjusted to 200 ng/mL with nuclease-free water. Ten microliters (μL) of clean RNA were reverse transcribed to cDNA using the cDNA reverse transcription kit from Applied Biosystems, Foster City, CA, USA and a gradient thermocycler from Eppendorf, Hauppauge, NY, USA. The cDNA synthesis cycle was set to 25 °C (10 min), 37 °C (120 min), 85 °C (5 min) and hold at 4 °C. The concentration of the cDNA synthesized was taken by the Nanodrop and adjusted to 20 ng/μL with nuclease-free water. Samples were constantly stored at −85 °C.

Gene expression was determined via RT-qPCR using StepOnePlus (Applied Biosystems, Carlsbad, CA, USA). Each reaction consisted of cDNA (1 μL), forward primer (0.6 μL), reverse primer (0.6 μL), nuclease-free water (7.4 μL), and SYBR Green (10 μL), giving a total of 20 μL of Master Mix (Applied Biosystems, Carlsbad, CA, USA). All samples had 3 replicates per gene on the same plate. The conditions for RT-PCR were set for holding stage at 50 °C (120 s), 95 °C (120 s); cycling stage at 95 °C (15 s) repeated for 40 cycles, and 60 °C (60 s); for melt curve stage at 95 °C (15 s), 60 °C (60 s) and finally 95 °C (15 s). At the end of the cycles, a melting temperature curve was determined. The mRNA expression of the following genes was determined: sodium-glucose linked transporter 1 (SGLT1), glucose transporter protein type 1 (GLUT1), glucose transporter protein type 2 (GLUT2), glucose transporter protein type 5 (GLUT5), glucose transporter protein type 8 (GLUT8), glucose transporter protein type 10 (GLUT10), and glucose transporter protein type 12 (GLUT12). The housekeeping gene used was beta-actin and data obtained were analyzed using the 2^−ΔΔCT^ method [28] with the TN0 group as the control for the other groups. The primer pairs used for RT-qPCR analysis is displayed in Appendix A.

### 2.6. Statistical Analysis

The performance and mRNA expression data were analyzed using the following statistical model.

$$y_{ijk}=\mu +\alpha_{i}+\beta_{j}+{\left(\alpha \beta \right)}_{ij}+\varepsilon_{ijk}$$

$where\;y_{ijk}=performance\;or\;mRNA\;expression\;of\;broiler\;chickens,$ μ is the overall mean of the response, $\alpha_{i}$ is the temperature effect, where i = 1, 2; $\beta_{j}$ is the effect of glucose, where j = 1, 2; ${\left(\alpha \beta \right)}_{ij}$ is the interaction effect between temperature and glucose (class *i* and *j*), and the $\varepsilon_{ijk}$ is the random error. The statistical model was implemental by a two-way ANOVA using SAS^®^ Studio software version 9.4 (SAS Institute Inc., Cary, NC, USA) and analyzed using the generalized linear model (GLM) procedure [29]. Tukey-HSD test was conducted for multiple comparison between treatment groups and the significant level was set at *p* < 0.05.

## 3. Results

The Metabolizable energy (ME) intake of broiler chickens raised with/without glucose supplementation under thermoneutral (TN) or cyclic heat stress (HS) conditions is presented in Table 1. Under each temperature condition (TN/HS), the groups with glucose supplementation had a higher (*p* < 0.05) ME intake when compared to groups without glucose supplementation.

Table 2 shows the effect of glucose supplementation on the performance characteristics of broiler chickens raised under TN or HS conditions. Body weight (BW) was similar (*p* > 0.05) at day 28 prior to initiating HS. Cyclic heat treatment resulted to an increase (*p* < 0.05) in FCR and WCR, while a decrease in the FI, BWG, and BW at day 35 when compared to birds raised under the TN condition. The birds supplied with glucose water had a significant reduction (*p* < 0.05) in FCR and FI relative to birds raised without glucose supplementation. The HS0 group had a significantly lower (*p* < 0.05) BW at day 35 and BWG, and a significantly higher (*p* < 0.05) FCR when compared to TN0 and TN6 groups. The FI and BWG observed in the HS6 group were lower (*p* < 0.05) relative to TN0 and TN6. Interestingly, the FCR of HS6 was similar (*p* > 0.05) to TN0 birds. HS0 had a higher (*p* < 0.05) water intake compared to TN6. Glucose supplementation reduced (*p* < 0.05) feed intake in both TN6 and HS6 birds relative to TN0 and HS0 groups, respectively.

The carcass yield of broiler chickens supplied with glucose under TN or HS conditions is shown in Table 3. Both cyclic heat treatment and glucose supplementation had no significant effect (*p* > 0.05) on *P. minor*-, thigh-, and wings-yields. Cyclic heat treatment significantly (*p* < 0.05) reduced eviscerated weight in the HS0 birds when compared to birds in TN0, TN6 and HS6. The abdominal fat was significantly increased (*p* < 0.05) in the HS group when compared to the TN group. There was a significant reduction (*p* < 0.05) in the live weight, eviscerated weight, percentage *P. major*, and drumstick of HS0 birds compared to TN0 and TN6. Glucose supplementation resulted in an increase (*p* < 0.05) in *P. major* yield and eviscerated weight of the birds.

**Figure 1:**
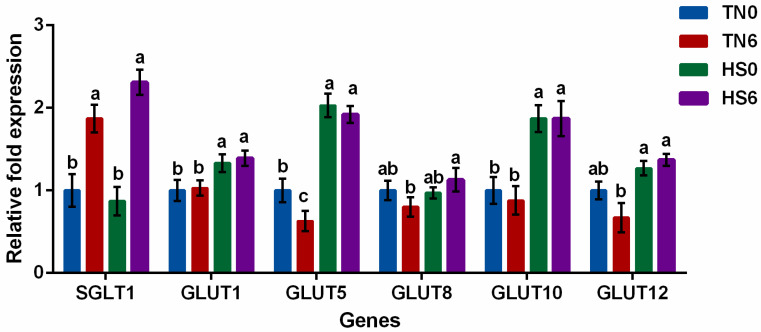
The expression of glucose transporters in the *Pectoralis major* (n = 6/treatment) of broiler chickens raised under thermoneutral or heat stress conditions (day 28–35). TN0—Thermoneutral (25 °C) without glucose supplementation, TN6—Thermoneutral (25 °C) with 6% glucose supplementation, HS0—Heat stress (25 °C–35 °C–25 °C) without glucose supplementation, HS6—Heat stress (25 °C–35 °C–25 °C) with 6% glucose supplementation. SGLT1—sodium glucose cotransporter 1, GLUT1—glucose transporter type 1, GLUT5—glucose transporter type 5, GLUT8—glucose transporter type 8, GLUT10—glucose transporter type 10, GLUT12—glucose transporter type 12. Error bars represent SEM. Superscripts ^a,b,c^ are used to indicate significant differences (*p* < 0.05) between treatments.

The mRNA expressions of glucose transporters in *P. major* of birds raised under TN or HS conditions are shown in Figure 1. Groups supplemented with glucose (TN6 and HS6) had a higher (*p* < 0.05) expression of SGLT1 compared to groups without glucose supplementation (TN0 and HS0). However, the expression of GLUT1, 5, and 10 were higher (*p* < 0.05) in HS groups compared to TN groups. GLUT5 was downwardly expressed (*p* < 0.05) in TN6 relative to TN0, HS0 and HS6 birds. Birds in HS6 had a higher expression of GLUT1, 5, 8, 10 and 12 when compared to birds in TN6 while GLUT 1, 5, and 10 were upwardly expressed in HS0 relative to TN0.

## 4. Discussion

Birds generally stop feeding when they meet their energy requirement. Therefore, it is expected that birds will reach their energy requirement earlier when fed a high energy diet compared to low energy died. In the current study, regardless of the heat treatment, chickens that received glucose supplementation reduced their feed intake. The chickens reared under either TN or HS conditions and received glucose supplementation consumed about 600 Kcal/g ME from glucose. The TN6 chickens reduced their feed intake by 9.5% while the HS6 reduced their feed intake by 13.1%. However, the glucose supplementation improved the total energy intake by 4.9 and 3.2% in the TN6 and HS6 birds, respectively. Glucose supplied through water is readily available energy compared to exogenous generation of glucose from feed. Exogenous generation of glucose via digested feed invariably generates heat which forms part of the heat load of chickens reared under HS. Therefore, using glucose supplementation through water during HS may have the capacity to reduce the endogenous heat load.

Nevertheless, HS reduced growth, feed intake and increased FCR and WCR. This is in concordance with several other HS-related studies [2,30,31]. Water is an important nutrient that birds need to maintain their thermal homeostasis when under heat stress. Although a significant difference in water intake (WI) was only observed between HS0 and TN6, the highest numerical value for WI was recorded in the HS0 group. The extra water intake of these birds is possibly a compensatory mechanism for water loss during thermoregulation under HS. This was reflected in the significantly high WCR ratio in the heat stressed birds compared with the TN birds. However, glucose supplementation did not significantly affect water intake nor WCR.

Birds supplemented with glucose had improved growth but the change was not significant. The non-significant difference between the final BW of the HS6 and TN0 did not reflect in the body weight gain. However, it appears that glucose supplementation reduced feed intake and improved FCR. Glucose supplementation significantly improved eviscerated weight of chickens reared under HS. There were no differences in eviscerated weight in the HS6 group compared with the TN groups. There was about 140 g differences in the eviscerated weight between the HS0 and HS6 groups, which represents about 9.2% improvement.

Whereas HS did not affect carcass-, *P. minor*-, thigh-, and wing-yield, it significantly increased abdominal fat yield and reduced *P. major*- drum stick-yield. Glucose supplementation significantly improved *P. major* yield, but did not affect the other part-yield measurements. Most importantly, the chickens reared under HS and supplemented with 6% glucose had a significantly higher *P. major* yield compared to their counterpart with no supplementation. The increase in *P. major* yield in the TN6 group was 14.2% higher than those in the TN0 group. The US alone produces over 9 billion of chicken for meat in 2018 [32] and this has been on the increase due to higher demands [33]. With persistent heat waves during the summer months, salvaging about 9% evisceration weight and 14% *P. major* yield through glucose supplementation is of significant economic importance.

The reduction in growth during HS is a combination effect of reduced feed intake resulting on concomitant reduction in protein synthesis and increase in protein catabolism [22,34]. Protein catabolism under HS may be the result of protein misfolding and aggregation in the endoplasmic reticulum [35] and gluconeogenesis [36]. Protein biosynthesis has been shown to be affected by glucose metabolism [35,37]. Therefore, it is possible that the glucose supplementation improved protein synthesis in the chickens reared under HS.

Breast meat yield is the most severely impacted variable in broiler chickens under HS. In the current study, the *P. major* yield is also the most positively impacted under glucose supplementation. Judging from the body weight gain, eviscerated carcass weight and *P. major* yield, it appears that glucose supplementation may have shifted protein synthesis from offal (internal organs) to other yield parameters, especially the *P. major*.

Heat stress increased relative fat deposition in broilers. Fat deposition is the resultant outcome between energy intake and utilization in birds [38], hence, any alteration in glucose metabolism will affect fat deposition in birds. Under HS, gluconeogenesis is favored, and Acetyl CoA carboxylase (ACC) is inhibited. This results in the production of more malonylcoA, thereby increasing fat deposition in HS birds.

We further evaluated how major glucose transporters are regulated in the *P. major* of birds subjected to TN or HS conditions with or without glucose supplementation. Movement (locomotion) and thermogenesis (regulating body temperature) are the two main purposes of skeletal muscle in birds [39]. Among the body’s organs, the skeletal muscle has the highest mass, accounting for 80% of postprandial glucose uptake from circulation, hence, aiding glucose homeostasis. Skeletal muscle is vital for the uptake of glucose (primary energy source) and the breast muscle, which consists primarily of the *P. major* and *P. minor*, is a prominent part of it. Glucose is transported mainly via sodium-dependent glucose transporter (SGLT) and glucose transporters (GLUTs) in epithelial and nonepithelial cells, respectively [40]. Sodium-dependent glucose transporter 1 (SGLT1) utilizes electrochemical gradient of sodium to move sugar molecules into cells in a manner that is against chemical gradients. D-glucose absorption into endothelial cells in the skeletal muscle may be mediated by SGLT1, localized in the luminal membrane of capillary endothelial cells, and exit via the basal plasma membrane, possibly containing a GLUT-type transporter [41]. The higher expression of SGLT1 in the *P. major* of TN6 and HS6 birds relative to TN0 and HS0 was due to the additional glucose supplementation. This provided extra glucose that was transported by SGLT1 (the main active glucose transporter) in the *P. major*, hence, increasing the expression of SGLT1. It has been reported that a diet with high level of glucose and sodium can orchestrate a higher expression of SGLT1 [42,43]. Glucose transporter protein type 1 (GLUT1) can aid the uptake of basal glucose or may serve as an exit route for glucose in cells [44]. Since basal glucose uptake for maintenance is expected in all groups, the higher expression of GLUT1 in HS group could mean that more glucose exited the *P. major* of the HS birds, probably due to body need for glucose in other parts of the body. Glucose transporter protein type 5 (GLUT5) facilitates the uptake of fructose, the higher expression of GLUT5 in the *P. major* of HS birds could be due to the birds trying to meet their energy requirement through fructose since glucose is in limited supply due to reduced feed intake. Hence, birds tried to utilize fructose in meeting the energy demand of the body. Glucose transporter protein type 10 (GLUT10) transports basic sugars while maintaining glucose level [45]. The increased expression of GLUT10 in HS birds could putatively aid in the maintenance of glucose level under HS. Heat stress stimulates insulin [46]. GLUT8 and 12 are known to be insulin sensitive [47], hence the increase observed in HS group relative to TN6 could be due to heat stress stimulated insulin activity.

## 5. Conclusions

Persistent heat exposure results in chronic HS, and impairs the performance of broiler chickens. Heat stress impairs growth and feed efficiency with significant economic losses. Herein, we supplemented the drinking water of chickens reared under either thermoneutral and heat stress with glucose. In the current study, 6% glucose supplementation led to about 3.2% increase in total energy intake of broiler chickens reared under HS. Glucose supplementation improved eviscerated carcass weight by 9.2% and major breast yield by 14.2%. We posit that the glucose supplementation may have improved the cellular glucose level which is usually reduced under HS. We also observed that glucose supplementation regardless of the heat condition increased the mRNA expression of SGLT1 transporter. Increase in SGLT1 transporter expression has been associated with increased cellular glucose intake. Thus, the improvements in some performance parameters under HS when glucose was supplemented could be attributed to some improvements in glucose metabolism.

## Figures and Tables

**Table 1:** Estimated metabolizable energy (ME) intake of broiler chickens raised with/without glucose supplementation under thermoneutral or heat stress conditions (day 28–35).

Treatment	Metabolizable Energy Intake (ME), kcal/Bird		
	Feed	Glucose Water	Total
TN0	4113.47	0	4113.47
TN6	3721.34	595.98	4317.32
HS0	3673.09	0	3673.09
HS6	3192.47	598.15	3790.62

ME from feed and glucose is 3125 kcal/kg and 4 kcal/g, respectively. TN0—Thermoneutral (25 °C) without glucose supplementation, TN6—Thermoneutral (25 °C) with 6% glucose supplementation, HS0—Heat stress (25 °C–35 °C–25 °C) without glucose supplementation, HS6—Heat stress (25 °C–35 °C–25 °C) with 6% glucose supplementation.

**Table 2:** Performance characteristics of broilers raised with/without glucose supplementation under thermoneutral (TN) or heat stress (HS) conditions (day 28–35).

Treatment	Body Weight at Day 28 (g/Bird)	Body Weight at Day 35 (g/Bird)	Body Weight Gain (g/Bird)	Feed Intake (g/Bird)	Feed Conversion Ratio	Water Intake (mL/Bird)	Water Conversion Ratio
TN0	1630.35	2220.96 ^a^	590.61 ^a^	1316.31 ^a^	2.30 ^bc^	2585.38 ^ab^	4.59 ^bc^
TN6	1700.32	2318.85 ^a^	618.53 ^a^	1190.83 ^b^	1.94 ^c^	2483.11 ^b^	4.05 ^c^
HS0	1639.76	1990.19 ^b^	350.43 ^b^	1175.39 ^bc^	3.41 ^a^	3443.72 ^a^	8.30 ^a^
HS6	1681.98	2066.69 ^ab^	384.71 ^b^	1021.59 ^c^	2.84 ^ab^	2492.28 ^ab^	6.95 ^ab^
SEM	18.6194	53.3621	41.5027	51.7322	0.202	326.3200	0.7424
*p* value	0.2034	0.0016	0.0004	0.0113	0.0004	0.0441	0.0019
Temperature							
TN	1665.33	2269.91 ^a^	604.57 ^a^	1252.25 ^a^	2.12 ^b^	2534.24	4.32 ^b^
HS	1660.87	2028.44 ^b^	367.57 ^b^	1113.90 ^b^	3.13 ^a^	2968.03	7.62 ^a^
SEM	18.6001	37.7718	29.3468	38.3656	0.143	225.8314	0.5249
*p* value	0.8674	0.0003	<0.0001	0.0104	<0.0001	0.1869	0.0003
Glucose							
0%	1634.63	2105.58	470.52	1245.85 ^a^	2.86 ^a^	3014.55	6.45
6%	1691.98	2192.77	501.62	1106.21 ^b^	2.39 ^b^	2487.72	5.50
SEM	18.6001	37.7718	29.3468	38.3656	0.1430	225.8314	0.5026
*p* value	0.0572	0.1148	0.4841	0.0197	0.0332	0.1175	0.2173

Superscripts ^a,b,c^ are used to indicate significant differences (*p* value < 0.05) between parameters across treatments (n = 6/treatment). TN0—Thermoneutral (25 °C) without glucose supplementation, TN6—Thermoneutral (25 °C) with 6% glucose supplementation, HS0—Heat stress (25 °C–35 °C–25 °C) without glucose supplementation, HS6—Heat stress (25 °C–35 °C–25 °C) with 6% glucose supplementation.

**Table 3:** Carcass yield of broilers raised under thermoneutral or heat stress conditions with/without glucose supplementation (day 28–35).

Treatment	Eviscerated Weight * (g)	Carcass Yield (%)	Abdominal Fat (%)	*P. major* (%)	*P. minor* (%)	Thigh (%)	Drumstick (%)	Wings (%)
TN0	1675.78 ^a^	72.62	0.93	17.57 ^a^	3.41	11.82	10.31 ^a^	7.22
TN6	1682.11 ^a^	72.71	0.83	17.93 ^a^	3.50	11.62	10.01 ^a^	7.36
HS0	1514.56 ^b^	73.59	1.21	14.84 ^b^	3.09	10.96	8.78 ^b^	6.84
HS6	1654.35 ^a^	73.77	1.22	16.94 ^a^	3.25	11.86	9.77 ^ab^	7.18
SEM	33.0543	0.6576	0.1287	0.5295	0.1570	0.3191	0.3352	0.1529
*p* value	0.0015	0.9531	0.7433	0.0005	0.2701	0.1701	0.0129	0.1062
Temperature								
TN	1678.94 ^a^	72.66	0.88 ^b^	17.75 ^a^	3.46	11.72	10.16 ^a^	7.29
HS	1573.83 ^b^	73.68	1.22 ^a^	15.89 ^b^	3.17	11.41	9.24 ^b^	7.01
SEM	23.3729	0.4650	0.0910	0.3744	0.1110	0.2257	0.2370	0.1081
*p* value	0.0051	0.1270	0.0116	0.0007	0.0738	0.3305	0.0100	0.0682
Glucose								
0%	1595.17 ^b^	73.11	1.07	16.20 ^b^	3.25	11.39	9.55	7.03
6%	1668.23 ^a^	73.24	1.02	17.44 ^a^	3.38	11.74	9.86	7.27
SEM	23.3729	0.4650	0.0910	0.3744	0.1110	0.2257	0.2370	0.1081
*p* value	0.0308	0.8389	0.6921	0.0254	0.4164	0.2744	0.3108	0.1178

Superscripts ^a,b^ are used to indicate significant differences (*p* < 0.05) between parameters across columns. * Head, giblets, shanks and visceral not included. TN0—Thermoneutral (25 °C) without glucose supplementation, TN6—Thermoneutral (25 °C) with 6% glucose supplementation, HS0—Heat stress (25 °C–35 °C–25 °C) without glucose supplementation, HS6—Heat stress (25 °C–35 °C–25 °C) with 6% glucose supplementation.

## Data Availability

The data were obtained during the study and are not available to the public at this time.

## Appendix A. Primer Pairs Used for RT-qPCR Analysis of *P. major* Glucose Transporters Gene Expression Levels

**Description**	**Gene**	**GeneBank Accession Number**	**Product Size (bp)**	**Align.**	**Primer Sequence**
Glucose transporters	SGLT1 (SLC5A1)	NM_001293240	97	Forward	5′GAGGAGAAACCCGATGAAAGAG3′
				Reverse	5′CTAAGCCACAGAACCAGTTGTA3′
	GLUT1 (SLC2A1)	NM_205209.1	105	Forward	5′CTTCTGCATACACTCCTTCTCC3′
				Reverse	5′TGGACGTGAAACCAGCTAAA3′
	GLUT8 (SLC2A8)	AB083371	309	Forward	5′GCAGCAGAGGTTATTCGCGCC3′
				Reverse	5′GCCTCCCAGTATTCCTCCAGC3′
	GLUT10 (SLC2A10)	XM_417383.5	133	Forward	5′CCGCTGCAGATGAGGTATTT3′
				Reverse	5′GTTTCTTCTCAGAGCCGTAGTG3′
	GLUT12 (SLC2A12)	XM_419733.5	110	Forward	5′AGAGAGTGGGGAGGTTCCC3′
				Reverse	5′TCAGGACGAGCCAAGACA3′
Fructose transporters	GLUT2 (SLC2A2)	NM_207178.1	577	Forward	5′ATGCTGGTGGTCAATGTCCTCTC3′
				Reverse	5′TGATGCCTGAGAACTGCTGCGAT3′
	GLUT5 (SLC2A5)	XM_417596.6	108	Forward	5′AGGCTGATCTCTGCCTTTG3′
				Reverse	5′GTCGATGTAGGTTCGGTTGTAG3′
B-actin		NM 205518.1	125	Forward	5′AGACATCAGGGTGTGATGGTTGGT3′
				Reverse	5′TCCCAGTTGGTGACAATACCGTGT3′
